# Avoidable hospitalization after family physician and rural health insurance: interrupted time series and regression analyses, Tehran province, Iran

**DOI:** 10.1017/S1463423618000300

**Published:** 2022-02-24

**Authors:** Sedigheh Salavati, Arash Rashidian, Hanan Hajimahmoodi, Sara Ememgholipour, Vida Varahrami, Elham Khodayarimoez

**Affiliations:** 1 Ph.D. Student, Department of Health Management and Economics, School of Public Health, Tehran University of Medical Science, Tehran, Iran; 2 Professor, Department of Health Management and Economics, School of Public Health, Tehran University of Medical Science, Tehran, Iran; 3 Doctor, Director General of Family Physician Program, Iran Health Insurance Organization, Tehran, Iran; 4 Associate Professor, Department of Health Management and Economics, School of Public Health, Tehran University of Medical Science, Tehran, Iran; 5 Associate Professor, Department of Economics, School of Economics and Political Sciences, Shahid Beheshti University, Tehran, Iran; 6 Ph.D. Student, School of Public Health, University of Alberta, Alberta, Canada

**Keywords:** avoidable hospitalization, family physician, interrupted time series analysis, rural health insurance

## Abstract

**Background:**

Studying the effect of primary health care development when simultaneously implemented with health insurance schemes assesses effectiveness and use of health care services and gives us insight on how to develop such interventions in different countries.

**Aim:**

To analyze the impact of health insurance and the family physician program on total hospitalizations, and the relation between avoidable hospitalizations and access to family physicians among the rural population in Iran.

**Methods:**

We conducted an interrupted time series (ITS) analysis of monthly hospitalization rates between the years of 2003 and 2014 to assess the immediate and gradual effects of these reforms on total hospitalization rates in the rural areas of Tehran province. In addition, we used a sample of 22 570 hospitalizations between 2006 and 2013 to develop a logistic regression model to measure the association between access to a family physician and avoidable hospitalizations.

**Findings:**

ITS analysis showed that there was an immediate increase of about 1.96 hospitalizations per 1000 inhabitants (*P*<0.0001, CI=1.58, 2.34) hospitalization rates after the reforms. This was followed by a significant increase of about 0.089 per 1000 inhabitants (*P*<0.0001, CI=0.07, 0.1). Hospitalization increase continued up to four years after the policy implementation. Following that, hospitalization rates decreased among the rural population (a decrease of 0.066 per 1000, *P*<0.0001, CI=−0.084, −0.048). Studying the hospitalizations that occurred between 2006 and 2013 showed that there were 4106 avoidable hospitalizations from among a sample of 22 570 hospitalizations. Results of logistic regression models including gender, age and access to family physician variables showed that there was no statistical relation between access to a family physician and avoidable hospitalizations.

**Conclusion:**

Reforms had access effect and caused increased hospital services uses in people with unmet needs. Also the reforms did not decrease avoidable hospitalizations, and therefore had no efficiency effect.

## Background

Primary health care (PHC) is the foundation of health care delivery systems and its strength lies in providing access to needed and high-quality health care services (De Maeseneer *et al.*, 2007). Family physicians, as the first point of contact between the general population and the health care system, have an important role in strengthening PHC-based health systems (Atun, 2004; Van Lerberghe, 2008).

The effects of primary health care development on hospitalizations are ambiguous. On the one hand, primary health care activities can reduce avoidable hospitalizations by timely delivery of primary services. This is called the efficiency effect (Ricketts *et al.*, 2001; Dafny *et al*., 2005). It is necessary to mention that based on existing theories, avoidable hospitalizations (AHs) are an important indicator in measuring the quality or accessibility of PHC (Rosano *et al.*, 2013). On the other hand, primary health care developments can increase referral sensitive hospitalizations by identifying the unmet needs of population in the first stages of health care delivery. Increasing referral sensitive hospitalization is known as access effect (Ricketts *et al.*, 2001; Dafny and Gruber, 2005). Identifying primary health care-related interventions and their effect mechanism – especially when these are simultaneously accomplished with social protection schemes – is important and gives us insight on how to develop such interventions in different countries.

In Iran, in 2005, the family physician program was implemented in all rural areas and towns with a population of <20 000. As part of this reform, about 6000 physicians and 4000 midwives were added to the present PHC network during a time span of three years (Rashidian *et al.*, 2013). In the family physician program health teams – comprised of a family physician and midwives – were responsible for the health of the population under their coverage. They were also responsible for following up on the patients and following their referral to secondary health care services. Meanwhile, at the same time, the Iran health insurance organization (IHIO) was obliged to cover all rural residents by providing a rural health insurance package. These national reforms aimed to improve the access of the rural population to family physicians services and protect them against financial risk.

The initial aim of policy makers in the implementation of the family physician and health insurance program in Iran was restriction of costs by prevention of disease, reducing referral burdens and avoiding unnecessary services (Majdzadeh, 2012). Previous studies in Iran showed that family physician and rural health insurance reforms had access effect by increasing hospitalization services use among rural residents. However, the efficiency effect of these reforms was not explored (Rashidian *et al.*, 2013; Salavati *et al.*, 2018; 2019;) only a county level study showed the reforms decreased the avoidable hospitalizations but because of special characteristics of the rural population, the results of mentioned study cannot generalized (Salavati *et al.*, 2018; 2019). Therefore, we do not have any insight about the effect of reforms on avoidable hospitalizations and the nature of this effect.

In the present study, we first investigated the effect of reforms on hospitalization rates in the Tehran province, in Iran. We then investigated the association between family physician access and avoidable hospitalizations and the effect of demographic characteristics on this association in rural areas.

## Methods

### Design and setting

The family physician and rural health insurance reforms were implemented in April 2005 in all rural areas and cities with population up to 20 000 across the country. Our study setting was in Tehran province, and we studied any members of the rural population hospitalized in this province.

We conducted an interrupted time series analysis to investigate the effect of national reforms on total hospitalizations (hospitalizations for all conditions). We analyzed the monthly hospitalization rate two years before and eight years after the intervention (132 monthly observations, April 2003 to March 2014).

A retrospective longitudinal study was carried out based on a sample of 22 570 hospitalization data between 2006 and 2013 to explore the association between access to a family physician and avoidable hospitalizations. As there are no pre-determined AHs codes for Iran, we identified a list of 61 potential AHs codes based on the literature review (Caminal *et al.*, 2004; Purdy *et al.*, 2009; Freund *et al.*, 2013). We got some physicians opinions about accuracy of these codes also were used them in another study in Iran as AHs (Salavati *et al.*, 2018; 2019).

The avoidable hospitalization codes included in this study are for angina, asthma, cellulitis, congestive heart failure, convulsions and epilepsy, chronic obstructive pulmonary disease, dehydration and gastroenteritis, ear, nose and throat infections, gangrene, hypertension, Influenza and pneumonia, nutritional deficiency, other vaccine-preventable diseases, pelvic inflammatory disease, pyelonephritis, dyspepsia and other stomach function disorders and migraine/acute headache. Each of these diagnosis classifications comprises several AH codes, which result in a total of 61 codes.

In order to conduct ITS analysis we received the population data from the Statistical Center of Iran and the health insurance coverage and monthly hospitalization data from the IHIO. No sampling methods were used in this study, and all hospitalization rate data between April 2003 to March 2014 were included in the study.

As no comprehensive database was found for data extraction, the longitudinal study was conducted using various databases to obtain diagnostic codes, gender, age, length of stay and cost per each rural hospitalization. We extracted the diagnostic codes, age and sex of each hospitalization which had been registered, based on the insurance serial number of Tehran residents from Tehran Deputy of Iran Health Insurance Organization database. In the next step, we identified the national code number of each hospitalized patient based on their insurance serial number with the aid of a database developed by the IHIO. The patients’ geographical address was found using their national number. Eventually, we obtained a sample of 22 570 hospitalizations, after removing unclear and ambiguous data. In order to assess the level of access of the rural population to a family physician, we used the Health Networks Development Plan developed by the Ministry of Health and Medical Education. This plan shows the existing health care centers in each rural area. As some rural areas may not have a health care center, and family physicians are not available in all rural areas, we categorized rural areas based on their access to a family physician into four groups: (1) villages with rural primary health centers with the permanent presence of a family physician, (2) rural areas with ‘health houses’ and the temporary presence of family physicians, (3) rural areas with no health centers that are covered by villages with health houses and are known as ‘satellite villages’ and (4) rural areas without any health centers that are visited by mobile health teams including family physicians once or twice a month and are known as ‘remote villages.’ Villages with PHC centers have the highest, and remote villages have the lowest access to family physicians, respectively.

### Analysis

We conducted ITS analysis to assess the short- and long-term effect of reforms on the total hospitalization rate of rural residents. We defined each observation as the number of hospitalizations per month divided by the total population under coverage of the health insurance during the course of one year. This was done to counteract the population growth problem. The Augmented Dickey Fuller test showed that the rural hospitalization rate trend is a stationary series (test statistic: −4.75; values: −4, −3.44, −3.14; CI: 99%, 95%, 90%; *P*=0.0006). We used the Interrupted Time Series Analysis package of Stata version 13.0 for the analysis. We also used the Actest program to diagnose higher order auto-correlations in primary estimated ITS model. As higher auto-correlation was seen on 12 lags, we considered it in estimation of the final time series model. It is necessary to mention that auto-correlation of residuals on 12 lags in the basic model was … in order to the fact that hospitalizations was monthly and for 11 years consecutively. The observed 12 (month) lag was expected, as hospitalization patterns usually follow seasonal and sometimes monthly patterns.

The logistic regression model was developed to assess the probability of decreased AHs in response to increased access to a family physician. We included gender, age and access to a physician as independent variables in the model, and after development of the model we conducted some diagnostic tests. Linktest results showed no specification errors in the Logit model. Hosmer and Lemshow statistics showed Goodness-of-fit and there was no co-linearity between the independent variables.

Also, we conducted a *χ* ^2^ test to determine if there was a statistical relation between age, gender and type of hospitalization. We also conducted two independent samples test to explore the differences in average LOS and hospitalization costs between avoidable and unavoidable hospitalizations. It is worthy of note that hospitalization costs were adjusted based on the health care services price index for the first year of study and then were converted from Iranian Rials (IRR) into international dollars ($Int), based on purchasing power parities. All analyses were conducted by STATA version 13 and SPSS version 18 software.

## Results

Table 1 presents ITS model estimation. ITS analysis results showed that the initial total hospitalization rate was 1.63 per 1000 population with a small secular increase in hospitalization rate estimated at 0.017 per 1000 inhabitants per month (*P*<0.0001, CI: 1.51–1.74) (Table 1). We saw the first interruption in April 2006, one year after the start of the interventions and there was an immediate increase of about 1.96 hospitalizations per 1000 inhabitants (*P*<0.0001, CI: 1.58–2.34). The gradual monthly increase in hospitalization rates also shifted upward, and a further 0.089 hospitalization per 1000 inhabitants per month (*P*<0.0001, CI: 0.07–0.1) was observed, compared with the secular pre-interruption trend.

**Table 1:** Coefficients of segmented regression model for hospitalization rates before and after the interventions

Variables	Value (SE)	*t*	*P*	95%	CI
Initial hospitalization rate	1.629 (0.05)	28.74	<0.0001	1.51	1.74
Slope before intervention	−0.017 (0.002)	−7.58	<0.0001	−0.02	−0.01
Change in level after the intervention	1.964 (0.19)	10.17	<0.0001	1.58	2.34
Change in slope compared with before the intervention	0.089 (0.009)	8.99	<0.0001	0.07	0.1
Change in level after second interruption	−0.715 (0.29)	−2.44	0.01	−1.29	−0.13
Change in slope compared with before the second interruption	−0.066 (0.009)	−7.34	<0.0001	−0.08	−0.04

**Fig. 1:**
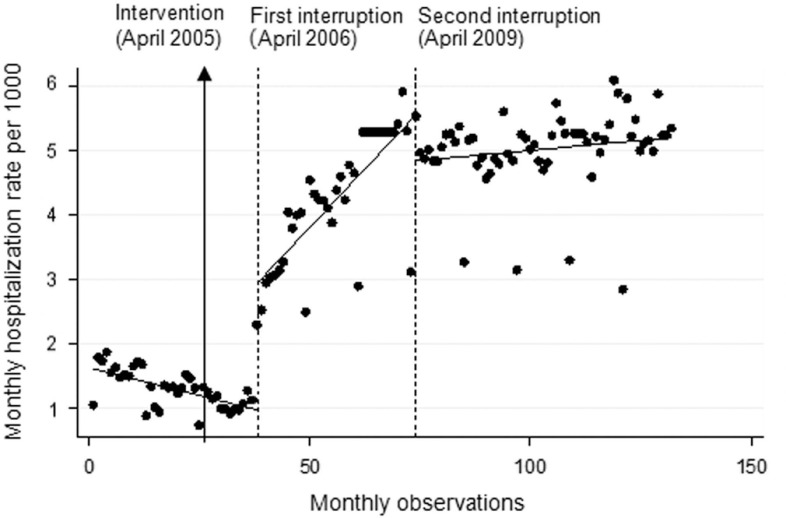
Interrupted time series analysis with Newey-West standard errors and 12 lags on rural hospitalizations rate

Identification of another interruption point three years after the first interruption (and four years after the decision to start the policy) demonstrated that the increase in hospitalization discontinued in April 2009. Results showed a decrease in hospitalization level at this point around 0.72 per 1000 inhabitants (*P*=0.01, CI: −1.29 to −0.13). A decrease in slope of about 0.066 per 1000 rural population is also witnessed beyond this second interruption point (*P*<0.0001, CI: −0.08 to −0.05) (Figure 1).

**Fig. 2:**
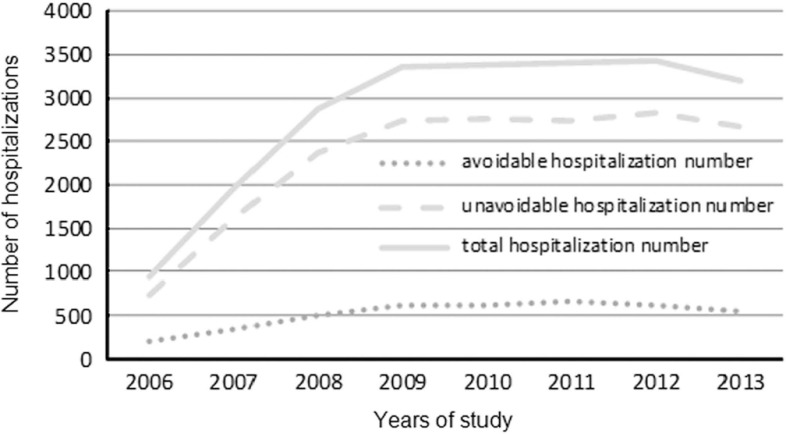
Number of hospitalizations after the family physician reform (2006–2013)

Analysis of hospitalizations between the years 2006 and 2013 showed that there were 4106 avoidable hospitalizations among a sample of 22 570 hospitalizations (18.2% of total hospitalizations). Both avoidable and unavoidable hospitalizations number showed an increase over the years of study, especially in the first three years (Figure 2).

Table 2 presents some characteristics of avoidable and unavoidable hospitalizations. The results of *χ* ^2^ test showed that avoidable and unavoidable hospitalization rates statistically varied among different age groups (*P*<0.0001, df=7, *χ* ^2^=1292). Hospitalization rates are also statistically different among men and women (*P*<0.0001, df=1, *χ* ^2^=89.139).

**Table 2:** Descriptive statistics of avoidable and unavoidable hospitalizations characteristics (2006–2013)

	Unavoidable hospitalization number	Avoidable hospitalization number	Statistics
Age			
1–10	2123 (74.23)	737 (25.77)	*P*=0 df=7 *χ* ^2^=1292
10–20	1451 (94.29)	88 (5.71)	
20–30	3230 (94.25)	197 (5.75)	
30–40	2638 (91.73)	238 (8.27)	
40–50	2450 (82.52)	519 (17.48)	
50–60	2188 (74.2)	761 (25.8)	
60–70	1354 (72.91)	503 (27.08)	
70<	1602 (69.02)	719 (30.98)	
Gender			
Female	9755 (84.16)	1835 (15.84)	*P*=0 df=1 *χ* ^2^=89.139
Male	8709 (79.32)	2271 (20.68)	
Access area			
Villages with PHC centers	6088 (82.8)	1265 (17.2)	*P*=0.009 df=3 *χ* ^2^=11
Villages with health houses	9817 (81.7)	2205 (18.3)	
Satellite villages	1648 (79.9)	415 (20.1)	
Remote villages	911 (80.5)	221 (19.5)	
Average length of stay (SD)	5.12 (7.73)	5.43 (5.44)	*t*=3.15 *P*=0.002
Average cost (SD)	1517 (3713)	1539 (3379)	*t*=0.37 *P*=0.71

The results of independent sample *t*-test showed that the average length of stay (*t*=3.15, *P*=0.002) in avoidable hospitalizations are higher than unavoidable hospitalizations. There was not any statistical difference between costs of avoidable and unavoidable hospitalizations.

Avoidable and unavoidable hospitalizations are significantly different in areas with different access to a family physician (*P*=0.009, df=3, *χ* ^2^=11) (Table 2).

The results of logit regression models are presented in Table 3. Based on Akaike information criterion model 2 was preferred. Based on model 2, there is no relation between access to a family physician and avoidable hospitalization. Also, the odds of avoidable hospitalizations in men are 15% higher than women. The elderly (over 70 years old) have higher avoidable hospitalizations compared with other age groups.

**Table 3:** The logistic regression model between access to family physician, gender, age and avoidable hospitalization

Models	Variables	Odds ratio (SE)	*Z*	*P*-value	95%	CI	Husmer Lemeshow statistics
Model 1	Gender						
	Men	1.38 (0.04)	9.4	0	1.29	1.48	*χ* ^2^ (4)=3.31 *P*=0.5
	Women (base)	–	–	–	–	–	
	Access						
	Remote villages	1.16 (0.09)	1.91	0.05	0.99	1.36	
	Satellite villages	1.20 (0.07)	2.92	0.004	1.06	1.36	
	Villages with health houses	1.08 (0.04)	2.20	0.02	1	1.17	
	Villages with health care centers (base)	–	–	–	–	––	
Model 2	Gender						
	Men	1.15 (0.04)	3.79	0	1.08	1.24	*χ* ^2^ (8)=12.18 *P*=0.14
	Women (base)	–	–	–	–	–	
	Age (years)						
	1–10	0.77 (0.04)	−4.17	0	0.68	0.87	
	10–20	0.13 (0.01)	−16.83	0	0.1	0.17	
	20–30	0.14 (0.01)	−22.69	0	0.11	0.16	
	30–40	0.2 (0.01)	−19.34	0	0.17	0.24	
	40–50	0.47 (0.03)	−11.22	0	0.41	0.54	
	50–60	0.77 (0.04)	−4.07	0	0.68	0.87	
	60–70	0.83 (0.05)	−2.63	0.009	0.72	0.96	
	70<	–	–	–	–	–	
	Access						
	Remote villages	1.1 (0.09)	1.12	0.26	0.92	1.31	
	Satellite villages	1.06 (0.07)	0.91	0.36	0.93	1.21	
	Villages with health houses	1.01 (0.04)	0.26	0.79	0.93	1.09	
	Villages with health care centers (base)	–	–	–	–	–	

## Discussion

While rural insurance and family physician policy was rolling out in Iran (and in Tehran province) in 2005, the policy effects on hospitalization rate were visible within a year after the initiation of the program. The increases in hospitalization trends continued up to three years after the first effect of reforms. In addition, we explored a sample of hospitalizations for the first seven years after the interventions and showed that both avoidable and unavoidable hospitalizations had grown during the period. This finding followed the pattern of increase in total hospitalization rates. We also noted that avoidable hospitalizations were not related to access to a family physicians, therefore we can say that the reforms did not have the expected efficiency effect through reducing avoidable hospitalizations.

These results are similar to the previous research conducted in Lorestan, a deprived province in Iran, in which the hospitalization rate slope increased until 40 months after the interventions but then returned back to the base line slope witnessed before the interventions. We can say that the reforms had a similar effect in both provinces. Even though Tehran as the capital of Iran is a non-deprived province, but the rural population encountered financial barriers in accessing hospitalization services before the interventions, and the reforms responded to the unmet needs of rural residents.

Based on a similar study that explored the effect of reforms across the country, the first effect was seen one year after the reforms implementation, and the increase in hospitalization continued up to 2 years after this first effect (Salavati *et al.*, 2017a; 2017b). Therefore the effect of reforms is visible in terms of access effect in both country and province level.

Studies that explored the effect of expanding the basic medical insurance schemes in China and the public health insurance of children, covered by Medicaid in the United States showed similar results (Dafny and Gruber, 2005; Wang *et al.*, 2014). Theoretically, the positive effect of health insurance expansion on the use and cost of health services and inpatient utilization has been approved (Buchmueller *et al.*, 2005). Therefore, it seems that developing universal health coverage in underserved areas results in higher hospitalizations that eventually lead to increased health care system expenditure that is in addition to the initial costs of reforms implementation in these health systems.

However, we should be cautious when talking about the efficiency effect of reforms in this study because in our study, the AHs comprised 22% of total hospitalizations after the reforms and showed a gradual decrease in the subsequent seven years, eventually amounting to 17% of total hospitalizations. This is seen alongside the increase of the life expectancy in Iran, and an increase in the burden of non-communicable diseases in recent years (Shahraz *et al.*, 2014). Therefore, if the percentage of AHs compared with total hospitalizations can remain fixed, this can itself be a sign of the efficiency effect of reforms.

Some previous study results are in confirmation of our results in terms of their being no relation between access to physicians and avoidable hospitalization. Physician supply in Colombia was not associated with AHs in rural areas, even though the negative relation was present in urban areas (Laditka *et al.*, 2005). Provision of chronic disease care services at the primary level for diabetes was associated with lower hospitalizations in London; however, this did not stand true for asthma (Saxena *et al.*, 2006). The supply level of physicians and their distribution had little effect on ambulatory care sensitive conditions in Medicaid beneficiaries in the United States (Krakauer *et al.*, 1996). Every preventive visit was related to an insignificant decrease in avoidable hospitalization risk among urban children in the United States (Steiner *et al.*, 2003).

Contrary to our results, a systematic review showed that adequate primary care physician supply and long-term relationships between physicians and patients reduce hospitalizations for chronic ambulatory care sensitive conditions (Van Loenen *et al.*, 2014). A study in Canada suggested that continuity of PHC is related with decreased ambulatory care sensitive conditions (Menec *et al.*, 2006). In Sweden, there was a negative relation between general practitioners’ visits and AHs (Lindström *et al.*, 2003). Also in Germany, AHs were related to the density of physicians in different areas (Freund *et al.*, 2013). With higher access to primary care, the continuity of care resulted in a decrease in preventable hospitalizations in Taiwan (Cheng *et al.*, 2010). Almost all of these studies were conducted in developed countries and considered the effect of physician supply on AHs. It seems some structural and technical characteristics of family physician program like number of physicians per population, continuity of care, presence of disease management guidelines and programs, etc. effect on this program outcomes. For example, the continuity of family physician care in rural areas in Iran and its effect on AHs is a matter that requires more study.

Some studies investigated the effect of introducing new PHC programs on AHs and presented results that were opposite to ours. For example, there was a negative relationship between being under coverage of a health maintenance organization and hospitalization for ambulatory care sensitive conditions among children in the United States (Friedman and Basu, 2001). The family health program coverage among children under five years of age had a protective effect against avoidable hospitalizations of these children in Brazil (Carvalho *et al.*, 2015). Based on diverse results we can say that the results of exploring the PHC expansion effect on AHs may differ according to the type of health-care system, type of investigated subjects and methods of analysis (Rosano *et al.*, 2013). Also, the quality of development and implementation of a new program can affect its outcome.

Our study results showed significantly higher AHs among men. Study on the effectiveness of community based and family-oriented primary health care programs in decreasing AHs showed that these programs were less effective among men (Guanais and Macinko, 2009). Gender has been introduced as a factor to affect patients’ behavior in insurance coverage-based utilization (Buchmueller *et al.*, 2005). In this study, it seems men were more often in a condition that led to hospitalization than women when referring to family physicians. This may be due to men visiting family physicians in rural areas less frequently because of their agricultural responsibilities.

In the present study, we found a higher length of stay and increased costs in avoidable hospitalizations. It can be noted that trying to decrease these types of hospitalizations can lead to decreased hospitalization costs. Therefore, the appropriateness of the primary care services provided by family physicians in rural areas should be the focus point of policy makers.

This study has several strengths. ITS designs are the strongest quasi-experimental designs to estimate intervention effects in nonrandomized settings and our study utilized this method in assessing the reforms effect on total hospitalization rates. We considered the effect of reforms on both total and avoidable hospitalizations; therefore the results present a comprehensive picture of the reforms effect and clarify both the access and efficiency effect of the reforms for policy makers. We explored the interventions effect with a large number of observations in ITS analysis and a large sample in investigating the association between access to physician and AHs. This allows for high power in testing the hypothesis and reaching reliable results.

## Limitations

The ITS analysis was carried out to assess the impact of reforms on total hospitalization rates. However, we did not use a control group to ensure that the immediate and long-term effects seen were due to these interventions, and no other factors or co-interventions were involved. Some variables such as socio-economic factors, the demographic characteristics of the population, co-morbidities and primary health care specifications are known factors that affect hospitalization (Goddard and Smith, 2001; Ansari, 2007). However, we did not consider these factors in exploring the association between access to a family physician and potentially avoidable hospitalizations. However, the exclusion of rural areas was indirectly considered in classifying rural areas into four groups based on their access to family physician. This is because in Iran, it can be said that rural areas with lower access to physicians have a lower economical and welfare status, although this cannot be confidently claimed.

## Conclusion

The family physician program and expansion of health insurance had an access effect and resulted in increased population use of hospital services, which may be due to identification of the unmet needs of population. The reforms did not have an efficiency effect through decreasing avoidable hospitalizations. In designing universal health coverage, policy makers should consider the expenditures that rise because of the use of more services. Also, items such as promoting appropriateness and effectiveness in using these services to contain the costs of avoidable hospitalizations should be taken into account. Although the rural areas included in the study were within Tehran Province, a province with the highest number of physicians and hospital beds per capita of population, we observed important unmet needs. These could have occurred due to financial barriers to access or uneven distribution of doctors and hospital beds within the province (Chavehpour *et al.*, 2017). Hence, a closer look at the distribution of doctors within the rural areas of the province is recommended. Rural areas in Tehran province should be treated similarly to other deprived areas of the country in terms of provision of access to medical care.

## References

[ref1] Ansari Z (2007) The concept and usefulness of ambulatory care sensitive conditions as indicators of quality and access to primary health care. Australian Journal of Primary Health 13, 91–110.

[ref2] Atun R (2004) *What are the advantages and disadvantages of restructuring a health care system to be more focused on primary care services?* Copenhagen: WHO Regional Office for Europe.

[ref3] Buchmueller TC, Grumbach K, Kronick R Kahn JG (2005) Book review: the effect of health insurance on medical care utilization and implications for insurance expansion: a review of the literature. Medical Care Research and Review 62, 3–30. 15643027 10.1177/1077558704271718

[ref4] Caminal J, Starfield B, Sánchez E, Casanova C Morales M (2004) The role of primary care in preventing ambulatory care sensitive conditions. The European Journal of Public Health 14, 246–251. 15369028 10.1093/eurpub/14.3.246

[ref5] Carvalho SC, Mota E Dourado I (2015) Hospitalizations of children due to primary health care sensitive conditions in Pernambuco State, Northeast Brazil. Cadernos de Saúde Pública 31, 744–754. 25945984 10.1590/0102-311x00069014

[ref6] Chavehpour Y, Rashidian A, Raghfar H, Emamgholipour sefiddashti S Maroofi A (2017) Seeking affluent neighborhoods? A time-trend analysis of geographical distribution of hospitals in the Megacity of Tehran. Health Policy and Planning 32, 669–675. 28453720 10.1093/heapol/czw172

[ref7] Cheng SH, Chen CC Hou YF (2010) A longitudinal examination of continuity of care and avoidable hospitalization: evidence from a universal coverage health care system. Archives of Internal Medicine 170, 1671–1677. 20937927 10.1001/archinternmed.2010.340

[ref8] Dafny L Gruber J (2005) Public insurance and child hospitalizations: access and efficiency effects. Journal of Public Economics 89, 109–129.

[ref9] De Maeseneer J, Willems S, De Sutter A, Van De Geuchte I Billings M (2007) Primary health care as a strategy for achieving equitable care. Health Systems Knowledge Network of the World Health Organization’s Commission on Social Determinants of Health.

[ref10] Freund T, Campbell SM Geissler S (2013) Strategies for reducing potentially avoidable hospitalizations for ambulatory care–sensitive conditions. The Annals of Family Medicine 11, 363–370. 23835823 10.1370/afm.1498 PMC3704497

[ref11] Friedman B Basu J (2001) Health insurance, primary care, and preventable hospitalization of children in a large state. American Journal of Managed Care 7, 473–488.

[ref12] Goddard M Smith P (2001) Equity of access to health care service: theory and evidence from the UK. Social Science & Medicine 53, 1149–1162. 11556606 10.1016/s0277-9536(00)00415-9

[ref13] Guanais F Macinko J (2009) Primary care and avoidable hospitalizations: evidence from Brazil. The Journal of Ambulatory Care Management 32, 115–122. 19305223 10.1097/JAC.0b013e31819942e51

[ref14] Krakauer H, Jacoby I, Millman M Lukomnik JE (1996) Physician impact on hospital admission and on mortality rates in the Medicare population. Health Services Research 31, 191. 8675439 PMC1070113

[ref15] Laditka JN, Laditka SB Probst JC (2005) More may be better: evidence of a negative relationship between physician supply and hospitalization for ambulatory care sensitive conditions. Health Services Research 40, 1148–1166. 16033497 10.1111/j.1475-6773.2005.00403.x PMC1361189

[ref16] Lindström K, Engström S, Bengtsson C Borgquist L (2003) Determinants of hospitalisation rates: does primary health care play a role? Scandinavian journal of primary health care 21, 15–20. 12718455 10.1080/02813430310000500

[ref17] Majdzadeh R (2012) Family physician implementation and preventive medicine; opportunities and challenges. International Journal of Preventive Medicine 3, ■.

[ref18] Menec VH, Sirski M, Attawar D Katz A (2006) Does continuity of care with a family physician reduce hospitalizations among older adults? Journal of Health Services Research & Policy 11, 196–201. 17018192 10.1258/135581906778476562

[ref19] Purdy S, Griffin T, Salisbury C Sharp D (2009) Ambulatory care sensitive conditions: terminology and disease coding need to be more specific to aid policy makers and clinicians. Public Health 123, 169–173. 19144363 10.1016/j.puhe.2008.11.001

[ref20] Rashidian A, Joudaki H, Khodayari-Moez E, Omranikhoo H, Geraili B Arab M (2013) The impact of rural health system reform on hospitalization rates in the Islamic Republic of Iran: an interrupted time series. Bulletin of the World Health Organization 91, 942–949. 24347733 10.2471/BLT.12.111708 PMC3845261

[ref21] Ricketts TC, Randolph R, Howard HA, Pathman D Carey T (2001) Hospitalization rates as indicators of access to primary care. Health & Place 7, 27–38. 11165153 10.1016/s1353-8292(00)00035-6

[ref22] Rosano A, Loha CA, Falvo R, van der Zee J, Ricciardi W, Guasticchi G Giulio de Belvis A (2013) The relationship between avoidable hospitalization and accessibility to primary care: a systematic review. The European Journal of Public Health 23, 356–360. 22645236 10.1093/eurpub/cks053

[ref23] Salavati S, Rashidian A, Emamgholipour S Varahrami V (2017a) The impact of rural health insurance and the family physician program on hospitalizations, a before-after study at the county level in Tehran province. *Medical Journal of The Islamic Republic of Iran* (in press).

[ref24] Salavati S, Rashidian A, Hajimahmoodi M, Emamgholipour S, Varahrami M Kheyr Andish M (2017b) Does rural health system reform affect hospitalization rates in Islamic Republic of Iran? An interrupted time series analysis. Manuscript submitted for publication.

[ref25] Saxena S, George J, Barber J, Fitzpatrick J Majeed A (2006) Association of population and practice factors with potentially avoidable admission rates for chronic diseases in London: cross sectional analysis. Journal of the Royal Society of Medicine 99, 81–89. 16449782 10.1258/jrsm.99.2.81 PMC1360495

[ref26] Shahraz S, Forouzanfar MH, Sepanlou SG, Dicker D, Naghavi P, Pourmalek F, Mokdad A, Lozano R, Vos T, Asadi-Lari M, Sayyari AA, Murray CH Naghavi M (2014) Population health and burden of disease profile of Iran among 20 countries in the region: from Afghanistan to Qatar and Lebanon. Archives of Iranian Medicine 17, 336. 24784862

[ref27] Steiner JF, Braun PA, Melinkovich P, Glazner JE, Chandramouli V, LeBaron CW Davidson AJ (2003) Primary-care visits and hospitalizations for ambulatory-care–sensitive conditions in an inner-city health care system. Ambulatory Pediatrics 3, 324–328. 14616042 10.1367/1539-4409(2003)003<0324:pvahfa>2.0.co;2

[ref28] Van Lerberghe W (2008) *The world health report 2008: primary health care: now more than ever*. World Health Organization.

[ref29] Van Loenen T, Van Den Berg MJ, Westert GP Faber MJ (2014) Organizational aspects of primary care related to avoidable hospitalization: a systematic review. Family Practice ■, cmu053.

[ref30] Wang S, Liu L, Li L Liu J (2014) Comparison of Chinese inpatients with different types of medical insurance before and after the 2009 healthcare reform. BMC Health Services Research 14, 1. 24382312 10.1186/1472-6963-14-1 PMC3880175

